# Is the Severity of the Clinical Expression of Anorexia Nervosa Influenced by an Anxiety, Depressive, or Obsessive-Compulsive Comorbidity Over a Lifetime?

**DOI:** 10.3389/fpsyt.2021.658416

**Published:** 2021-07-01

**Authors:** Elise Riquin, Agathe Raynal, Lama Mattar, Christophe Lalanne, France Hirot, Caroline Huas, Jeanne Duclos, EVHAN group, Nathalie Godart, Sylvie Berthoz, Nathalie Godart

**Affiliations:** ^1^Department of Child and Adolescent Psychiatry, Centre Hospitalier Universitaire d'Angers [Angers University Hospital], Angers, France; ^2^Laboratory of Psychology, LPPL EA4638, University of Angers, Angers, France; ^3^Adolescent and Young Adult University Hospital Department of the Health Foundation of Students of France, Centre Pierre Daguet, Sablé-sur-Sarthe, France; ^4^Department of Child and Adolescent Psychiatry, CH du Rouvray-CHU de Rouen, Rouen, France; ^5^Nutrition Division, Department of Natural Sciences, School of Arts and Sciences-Lebanese American University, Beirut, Lebanon; ^6^Université Paris Diderot [Paris Diderot University], Paris Sorbonne Cité, Paris, France; ^7^CESP, Univ. Paris-Sud, UVSQ, INSERM U 1178, Université Paris-Saclay [Paris-Saclay University], Paris, France; ^8^Department of Psychiatry for Adolescents and Young Adults, Institut Mutualiste Montsouris, Paris, France; ^9^Adolescent and Young Adult University Hospital Department of the Health Foundation of Students of France, Paris, France; ^10^Univ. Lille, CNRS, CHU Lille, UMR 9193—SCALab—Cognitive and Affective Sciences, Lille, France; ^11^Hôpital Saint Vincent de Paul, GHICL, Département de Psychiatrie, Paris, France; ^12^Univ. Bordeaux INCIA CNRS UMR 5287, Bordeaux, France; ^13^UFR des Sciences de la Santé Simone Veil [Simone Veil Health Science Training and Research Unit], Université de Versailles Saint-Quentin-en-Yvelines [Versailles Saint-Quentin-en-Yvelines University], Versailles, France

**Keywords:** anorexia nervosa, anxiety, depression, nutritional status, body mass index

## Abstract

**Purpose:** The relationship between anxiety or depressive comorbidities, their chronology of onset, and the severity of anorexia nervosa (AN) is not well-studied. We hypothesize that the existence of a comorbidity, particularly before the onset of AN, is associated with greater severity of AN.

**Methods:** One hundred seventy-seven subjects were assessed. The prevalence of major depressive disorder (MDD), obsessive-compulsive disorder (OCD), generalized anxiety disorder (GAD), and social phobia (SP) as well as their chronology of onset were studied. The assessment criteria of AN severity were the overall clinical condition, body mass index (BMI) on admission, lowest BMI, intensity of the eating symptoms, age at the onset of AN, illness duration, number of hospitalizations, and quality of life.

**Results:** Patients with AN had the greatest clinical severity when they had a comorbid disorder over their lifetime, such as MDD, GAD, or SP. These comorbidities along with OCD were associated with a higher level of eating symptoms and a more altered quality of life. A profile of maximum severity was associated with a higher prevalence of MDD and GAD. Concerning the chronology of onset, the age at the start of AN was later in cases of MDD or GAD prior to AN.

**Conclusion:** There seems to be an association between severity of AN and both MDD and GAD. The chronology of onset of the comorbidity did not seem to be associated with the severity.

## Introduction

Anorexia nervosa (AN) is a condition that involves restriction of calorie intake, leading to a significantly low weight, intense fear of gaining weight, and dysmorphophobia (DSM-5) (1). Its lifetime prevalence is estimated between 0.9 and 2.2% (2). It is a particularly severe condition (3). In fact, half of people treated for AN in adolescence recover from this disorder, but they can have other psychiatric disorders as 21% have chronic eating disorders (EDs) and 5% die (4). Of the deaths, around half are due to somatic complications and half to suicide. Over an entire lifetime, AN is associated with psychiatric comorbidities, such as major depressive disorder (MDD) in up to 64% of cases with at least one anxiety disorder in up to 72% of cases and with obsessive-compulsive disorder (OCD) in up to 62% of cases (5). The etiopathogenesis of AN remains poorly understood; the trajectories of vulnerability and clinical expression are heterogeneous (6, 7). Better characterization of the phenotypic subgroups of patients could clarify the etiopathogenesis and also differentiate the therapeutic strategies (8).

AN can be described by different degrees of clinical severity based on the overall clinical condition (divided by intensity of the malnutrition and severity of the ED) and its consequences (impact on social adaptation, illness duration, response to care) (9). These degrees of severity are also found within the forms requiring hospitalization. The intensity of the clinical severity in AN was shown to be prognostic of the outcome (10). Thus, the factors for a poor prognosis are a long illness duration, a premorbid developmental abnormality (including premorbid OCD), obsessive-compulsive personality disorder, the intensity of malnutrition over the course of progression, the subsequent occurrence of bulimia, and the use of purgatives (11).

The relationship between anxiety-depressive (AD) comorbidity and prognosis has been studied. Some authors show that AD comorbidity could have a negative impact on the overall outcome and the body mass index (BMI) of patients, and others have find the opposite (12). The relationship between AD comorbidity and the severity of clinical expression of AN in terms of overall clinical condition or eating symptoms has been studied very little. The only data in the literature show that psychiatric comorbidities (including AD disorders, OCD, and personality disorders) are associated more with re-hospitalizations (13). Furthermore, concomitant depression increases the suicidal risk in AN (14): premorbid OCD contributes to poor prognosis; social phobia and agoraphobia affect the subjects' quality of life (QOL) and increase ED symptoms 6–12 years after inpatient treatment (15). However, to our knowledge, no studies to date consider the relationship between the severity of current AN and the lifetime existence of psychiatric comorbidities.

None of the previously cited studies concerning the relationship between AD comorbidity and clinical severity of AN in the acute phase of the disorder took into account the relative chronology of AN and AD onset. Chronology is a potential contributory factor for explaining the different profiles of progression in AN. In fact, we were able to show that although the comorbidity of OCD over a lifetime in AN is not associated with a particular outcome, the existence of premorbid OCD before AN is a poor prognostic factor for the future. This did not hold true for MDD, whether it was preexisting or on a lifetime scale (15).

As a result, we conducted this study to test the hypothesis that the existence of an AD or OCD comorbidity during a lifetime, particularly when it started before the AN, is associated with a more severe overall clinical expression of AN in the acute phase of the disorder, including in each area of clinical expression (nutritional state, age at the start of AN, illness duration, number of hospitalizations, eating symptoms, and QOL).

## Methods

### Study Population

This study was part of a larger longitudinal multicentered study named EVHAN (Evaluation of Hospitalization for AN, Eudract number: 2007-A01110-53, registered in Clinical trials) between April 2009 and May 2011. The study protocol was approved by the Ile-de-France III Ethics Committee and the CNIL (Commission nationale de l'informatique et des libertés).

Inclusion criteria for the current study were as follows: being hospitalized for AN, admission BMI <15 and/or sudden and rapid weight loss, agreement to participate in the study, and being affiliated to the French Social Security health coverage system. Exclusion criteria were refusal to participate, insufficient command of the French language, existence of a potentially confounding pathology (e.g., diabetes, Crohn's disease, or other metabolic disorders), and being under the age of 13.

In accordance with the Helsinki declaration, written informed consent was obtained from each patient before inclusion and from the parents of those who were under 18 years old. Patients (if adults) or, in the case of children, both patients and their parents gave their written consent.

### Ethics Statement

This study is part of a larger longitudinal multicentered study named EVHAN (Evaluation of Hospitalization for AN, Eudract number: 2007-A01110-53, registered in Clinical trials). The study protocol was approved by the Ile-de-France III Ethics Committee and the CNIL (Commission nationale de l'informatique et des libertés). In accordance with the Helsinki declaration, written informed consent was obtained from each patient before inclusion and also from the parents for those who were under 18 years old.

**Figure 1:**
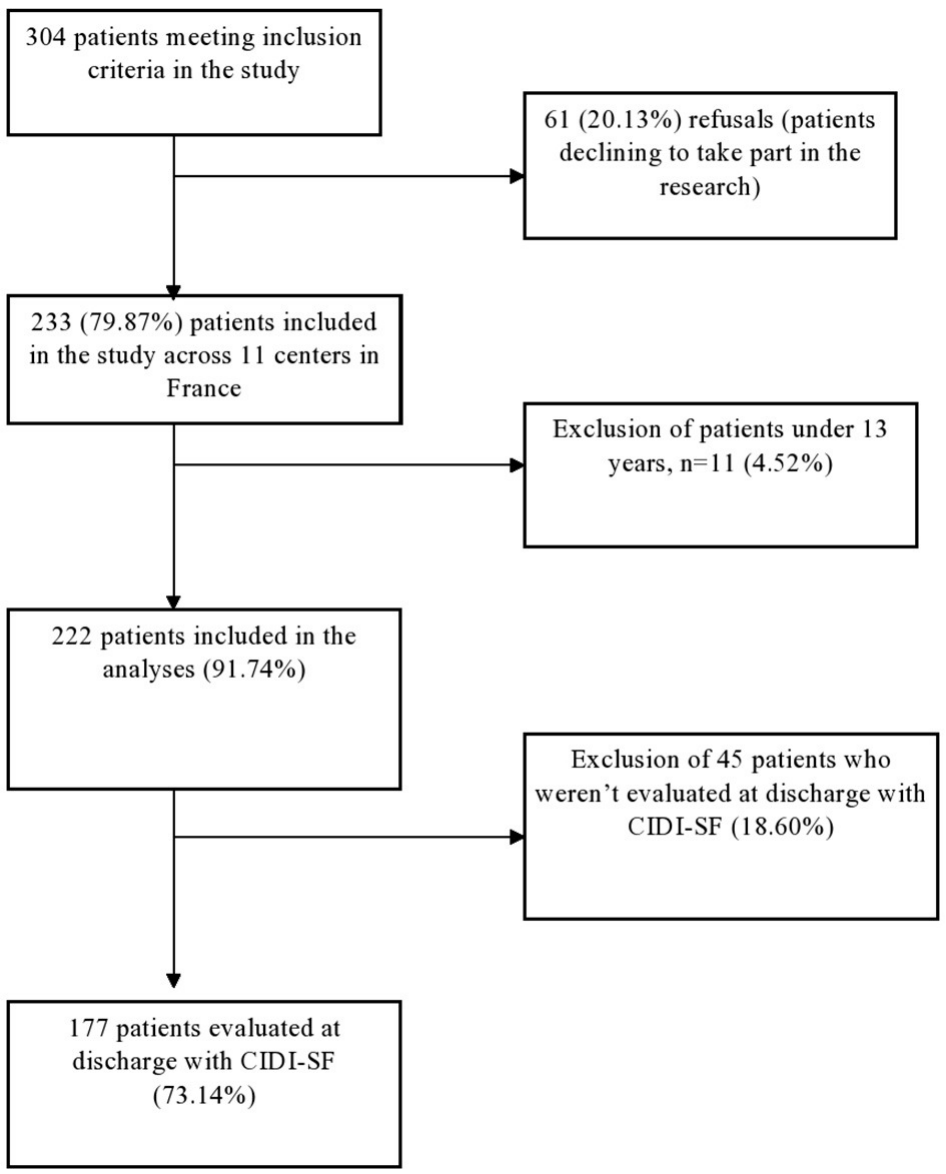
Flow chart. CIDI, Composite International Diagnostic Interview.

Sample patients were recruited from the inpatient treatment facilities of 11 centers in France (CHU-Bordeaux, Cochin—Maison des Adolescents, Institut Mutualiste Montsouris, MGEN—La Verrière, CHU-Nantes, CHU-Rouen, Robert Debre Hospital, Sainte-Anne Hospital, Saint-Etienne Hospital, Villejuif—Paul Brousse). Three hundred thirty-three consecutive patients with AN were involved in the EVHAN study between April 2009 and May 2011 (Figure 1). Inclusion criteria for the current study were as follows: being hospitalized for AN, admission BMI <15 and/or sudden and rapid weight loss, agreement to participate in the study, and being affiliated to the French Social Security health coverage system. Exclusion criteria were refusal to participate, insufficient command of the French language, existence of a potentially confounding pathology (e.g., diabetes, Crohn's disease, or other metabolic disorders), and being under the age of 13. In all, therefore, 222 patients (with full syndrome or subthreshold AN) were included in this study.

### Diagnosis of AN

A current diagnosis of AN was based on the DSM-IV-TR criteria (16). These criteria were assessed using the Eating Disorder Examination (EDE-Q v. 5.2) and the Composite International Diagnostic Interview Short Form (CIDI 3.0) (17–19) (Kessler). The CIDI Short Form is an easy-to-use tool derived from the CIDI (17, 20) and has been widely used ever since for epidemiological studies in Canada (21–23) and Europe (24–27) and compared with the CIDI (18, 23). It consists of a structured interview developed under the aegis of the World Health Organization (WHO) for epidemiological and clinical research purposes.

The BMI criterion was <10th percentile up to 17 years of age and BMI <17.5 for 17 years of age and above (28). After the exclusion of patients for whom response to the short CIDI were missing, the sample size was 177 patients. Participants were assessed during the first 2 weeks of hospitalization.

### Measures

The data were collected during the patient's hospitalization for AN. Each patient was selected by consecutive enrollment. Sociodemographic data and medical antecedents were collected, including age, age at AN onset, illness duration, number of hospitalizations for an ED, and duration of hospitalization. Nutritional status was assessed using the current BMI and the lowest BMI at the onset of the disorders.

Lifetime comorbidities (both current and/or past) were evaluated by questionnaires as follows:

The diagnoses of MDD, OCD, generalized anxiety disorder (GAD), and social phobia were made using the Composite International Diagnostic Interview Short Form (CIDI-SF) (18). Likewise, the chronology of onset was evaluated from the ages at AN onset, and each comorbidity was recorded with the CIDI-SF. The chronology of each onset was classified as follows: No comorbidity, comorbidity before AN onset, and comorbidity concomitant to or after AN onset.

The overall clinical condition was evaluated by the Global Outcome Assessment Scale (GOAS) by Morgan–Russell, a proxy questionnaire that assesses functioning over the past 6 months on five subscales exploring food, menstrual periods, mental state, psychosexual functioning, and socioeconomic status (28). It provides a score from 0 to 12. Lower scores indicate poorer clinical condition.

The severity of the ED symptoms was evaluated by the Eating Attitudes Test (EAT) (29, 30), which is a six-point format, self-report scale measuring a broad range of ED symptoms. It includes 26 items with three subscales: dieting, bulimia, and food preoccupation and oral control. It can range from 0 to 78 (maximum of symptoms) (29). Higher scores reflect a greater severity of ED symptoms.

QOL was assessed using the Eating Disorders Quality of Life (EDQOL), which is a 25-item self-reporting questionnaire, using four subscales (psychological, physical/cognitive, financial, professional/academic) (31). Possible ranges for each subscale and the total score are zero to four. Lower scores indicate a better QOL.

### Statistical Analyses

The data were processed on SPSS Statistics 19 package. A description of the sample was made using means, standard deviations, minimum, and maximum in the case of numerical variables and frequencies in cases of categorical variables. Chi-squared or Fisher exact tests were used for the categorical variables (i.e., comparison of prevalence between groups). The study of the relationship between AD comorbidity (categorical variable) and the continuous variables (overall clinical condition [GOAS], BMI, lowest BMI since the start of the disorder, intensity of the eating symptoms [EAT], age at AN onset, illness duration, number of hospitalizations and QOL [EDQOL]) was performed through comparison tests of means using Student's *t*-test. The study of relationships between the chronology of onset of the comorbidities in three classes (prior to AN, concomitant to or after AN, and absence) and the continuous variables was performed using ANOVA and then Student's *t*-test between groups when appropriate. The study of the relationships between comorbidities and their chronology of onset was carried out using Pearson's chi-squared test. Cluster analysis is a useful tool in the identification of subtypes. However, the selection of variables should be guided by a clearly stated theoretical framework in order to guide the interpretation of the results. According to the unsupervised approach, we primarily used an agglomerative (hierarchical) clustering procedure to partition the data set into subgroups whose characteristics might well-describe the typology observed through clinical inspection of the subjects. Hierarchical clustering using Ward's criterion and squared Euclidean distances were used to isolate homogeneous clusters of patients in order to delineate severity profiles based on the following variables: the overall psychosocial state of the subjects, assessed by five subscales of the GOAS (food, menstrual periods, mental state, psychosexual functioning, and socioeconomic status); symptoms of the eating disorder, assessed through three subscales of EAT (restriction, bulimia, and control); nutritional status, assessed through BMI on admission and minimum BMI; history of development of the disorder, assessed through age at AN onset, the illness duration, the number of hospitalizations; and their QOL, assessed through four subscales of the EDQOL (psychological, physical/cognitive, financial, professional/academic).

The study of the relationships between these various clusters and the presence or absence of an AD comorbidity and their chronology of onset was performed using Pearson's chi-squared tests.

The severity profiles were analyzed based on dendrograms derived from hierarchical clustering with a varying number of cluster solutions (two to six). Several solutions were then examined with the population of our sample combined into two to six clusters. Only the solutions with two and three clusters could be interpreted in terms of distribution of sample numbers and profile of means and were, therefore, selected and presented here.

A *p* < 0.05 was considered significant.

## Results

### Description of the Sample

#### Characteristics of the Population

The sample included in this study comprises 177 patients (Figure 1) (for description, see Table 1); 84 (47.5%) of them had AN restrictive type (AN-R), and 93 (52.5%) had AN binge eating/purging type (AN-BP). The sample was 96% female (170 women and seven men). The general condition of the patients was very severe as reflected in the total GOAS score, the mean of which was 4.8 (SD: 1.4). The subscales for the eating symptoms (A) and menstrual periods (B) had lower means with, respectively, 1.41 (SD: 1.7) and 2.5 (SD: 4.25), reflecting eating symptoms among the most severe and nearly constant amenorrhea. The mean for the mental state was 5.8 (SD: 1.7). The dimensions of psychosexual functioning (D) and economic status (E) had means, respectively, of 7.0 (SD: 2) and 7.0 (SD: 2.2). The mean score of the EAT questionnaire was 35.1 (SD: 16.8). Last, the EDQOL score was low with a mean score of 2.3 (SD: 0.6).

**Table 1:** Clinical characteristics of the sample (*n* = 177).

	**Average**	**SD**	**Minimum**	**Maximum**
Age at admission	20.5	6.2	13.2	51.2
BMI (kg/m^2^)	14.3	1.5	10.3	18.9^*^
minimum BMI (kg/m^2^)	13.1	1.5	8.6	18.5
Age at onset of AN	16.3	4.2	6	33
Illness duration (years)	3.9	4.1	0	24
Number of hospitalizations	2.6	4.1	0	37
Duration of hospitalization (weeks)	17.8	13.3	2	80

*BMI, Body Mass Index; SD, standard deviation*.

* *Initially overweight patient who received physical care before her entry into specialized care, diagnosis of AN effective in the 3 months before inclusion*.

#### Description of the Comorbidities and the Chronology of Onset Relative to AN

More than half of the sample had experienced MDD over their lifetime, close to one third had had GAD or social phobia, and more than one quarter OCD. According to PTSD symptoms, 13 patients (7.3%) had this diagnosis. We had no information on age of onset or end. Concerning the concomitant comorbidities of the ED, more than one third of patients had experienced GAD, about one quarter MDD, and social phobia and one fifth OCD. The results are detailed in Table 2.

**Table 2:** Psychiatric disorder comorbidities over a lifetime: prevalence and chronology of onset (*n* = 177).

	**Lifetime**	**Current**	**Before AN**	**Concomitant to or after AN**	Absent^*^	**χ^**2**^**	***p***	***Post-hoc***
MDD	101/177 (57.1%)	31/177 (30.7%)	42/177 (24.6%)	53/177 (31%)	76/177 (44.4%)	4.7	0.095	After=Before
OCD	48/177 (27.1%)	10/177 (20.8%)	22/177 (12.7%)	22/177 (12.7%)	129/177 (74.6%)	64.7	<0.001	Abs>Before; Abs>After
GAD	57/177 (32.2%)	22/177 (38.6%)	26/177 (15.3%)	24/177 (14.1%)	120/177 (70.6%)	49.7	<0.001	Abs>Before; Abs>After
Social phobia	57/177 (32.2%)	16/177 (28.1%)	35/177 (20.5%)	16/177 (9.4%)	120/177 (70.2%)	47.1	<0.001	Before>afterAbs>Before; Abs>After

*Before, comorbidity before the onset of AN; After, comorbidity after the onset of AN; Abs, absence of comorbidity; AN, Anorexia Nervosa; GAD, Generalized Anxiety disorder; MDD, Major Depressive Disorder; OCD, Obsessive Compulsive Disorder; PTSD, Post-traumatic Stress Disorder (PTSD)*.

* *No comorbidity and for some no information on the chronology*.

OCD and GAD, when they existed, occurred equally before and after AN (*p* < 0.0001). Social phobia, however, occurred more often before AN (*p* < 0.0001). There was no significant difference in the chronology of onset of MDD.

### Univariate Analyses

#### Comparison of Patients' Clinical Condition Based on the Existence of a Comorbidity

##### Severity of the Clinical Expression of AN: The GOAS Score

The results are described in the Table 3. When patients had experienced a comorbidity over their lifetime in terms of MDD, GAD, or social phobia, the clinical severity of the AN as measured by the GOAS score was significantly greater than in the case of no comorbidity at all (respectively, *p* < 0.0001, *p* = 0.004, *p* = 0.03). The difference was not significant for OCD.

**Table 3:** Comparison of average scores on clinical condition assessment scales according to the presence or absence of a comorbidity (per Student's *t*-test).

**Comorbidity**	**Disorder absent**	**Disorder present**	***t***	***p***
**Morgan-russell scale score (SD)**				
MDD	**5.3** (1.39)	**4.4** (1.34)	**4.2**	**<0.001**
OCD	4.8 (1.5)	4.7 (1.4)	0.5	0.6
GAD	**5.0** (1.4)	**4.33** (1.4)	**2.9**	**0.004**
Social phobia	**4.9** (1.4)	**4.4** (1.5)	**2.1**	**0.03**
**BMI (kg/m** ^**2**^ **)**				
MDD	14.5	14.1	1.4	0.2
OCD	14.3	14.3	−0.2	0.8
GAD	14.3	14.2	0.8	0.5
Social phobia	14.3	14.3	−0.1	0.9
**BMI (kg/m** ^**2**^ **)**				
MDD	13.4	12.9	2.2	0.3
OCD	13.4	13.0	1.0	0.3
GAD	**13.5**	**12.7**	**2.6**	**0.01**
Social phobia	13.1	13.2	−0.1	0.9
**Age of onset of AN**				
MDD	15.8	16.7	−1.3	0.2
OCD	16.4	16.1	0.4	0.7
GAD	16.1	16.8	−0.9	0.4
Social phobia	16.4	16.2	0.2	0.8
**Duration of progression (years)**				
MDD	3.2	4.4	−1.7	0.09
OCD	3.7	4.3	−0.8	0.4
GAD	**2.95**	**6.15**	–**4.48**	**0.000**
Social Phobia	3.68	4.27	−0.79	0.43
**Number of hospitalizations**				
MDD	**1.85**	**3.2**	–**2.1**	**0.037**
OCD	2.45	3.05	−0.81	0.42
GAD	**1.94**	**3.94**	–**2.98**	**0.003**
Social phobia	2.49	2.88	−0.55	0.58
**EAT**				
MDD	**30.76**	**38.42**	–**3.07**	**0.003**
OCD	**32.88**	**41.3**	–**3.02**	**0.003**
GAD	**32.71**	**40.16**	–**2.81**	**0.006**
Social phobia	**31.6**	**42.66**	–**4.27**	**0.000**
**EDQOL**				
MDD	**2.5**	**2.15**	**3.65**	**0.000**
OCD	**2.37**	**2.1**	**2.52**	**0.01**
GAD	**2.4**	**2.12**	**2.64**	**0.01**
Social phobia	**2.42**	**2.05**	**3.576**	**0.000**

*MDD, Major depressive disorder; OCD, Obsessive-compulsive disorder; GAD, Generalized anxiety disorder; EAT, Eating Attitudes Test; EDQOL, Eating Disorder Quality of Life. Bold values represents significant results*.

##### Other Aspects of the Clinical Expression of AN

All the studied comorbidities were significantly associated with a higher mean level of eating symptoms (EAT score) and a more altered QOL related to the ED (EDQOL score). GAD was associated with a significantly longer illness duration (*p* < 0.0001), more frequent hospitalizations before the current assessment (*p* = 0.003), and a poorer nutritional state over the course of the lifetime (minimum BMI) (*p* = 0.01). MDD was associated with more frequent hospitalizations (*p* = 0.037). BMI on admission and the age at AN onset did not differ significantly based on the existence of a comorbidity.

#### Comparison of Patients' Clinical Condition Based on the Chronology of Onset of the Comorbidity or Its Absence

The results are described in the Supplementary Table A. We found very few differences between the groups of patients with comorbid disorders prior to AN and those with later comorbid disorders. Most of the observed differences were between the groups without comorbidities and the groups with comorbidities before or after, which confirms the results shown in the preceding paragraph for both of these groups.

The chronology of onset of disorders only shows a significant difference for the age of AN onset, which was later in cases of MDD (17.9 years vs. 15.2 years, *p* = 0.009) and GAD (18.0 years vs. 14.3 years, *p* = 0.009) before AN.

Considering the sample size of PTSD and the absence of chronology data, no subsequent analyses were carried out.

### Hierarchical Classification

**Figure 2:**
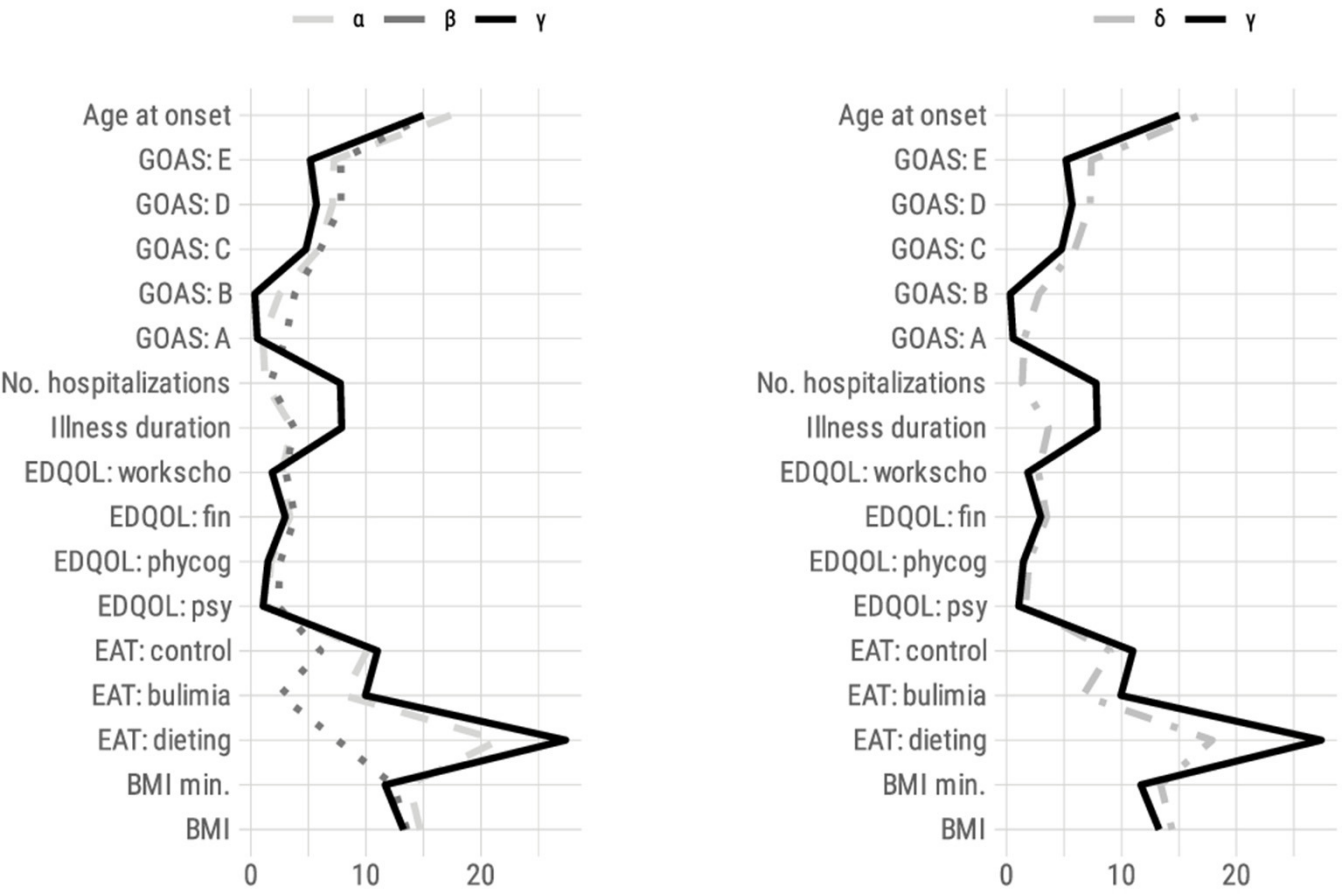
Profile of responses (mean) for all variables used in hierarchical clustering for each cluster solution (left: three clusters; right: two clusters). BMI, Body Mass Index; EAT, Eating Attitudes Test; EDQOL, Eating Disorder Quality of Life; psy, psychological; phy cog, physical/cognitive; end: financial; work scho, professional/academic; No: number; GOAS, Global Outcome Assessment Scale by Morgan-Russell.

Table 4 shows the results of applying agglomerative clustering based on squared Euclidean distance with Ward criteria. As can be seen from the dendrogram, a solution consisting of three clusters with *N* = 88, 31, and 24 subjects seems to be an appropriate way to represent our results. The clusters are described in Figure 2.

**Table 4:** Averages of variables representing the clinical severity of AN based on clusters.

**Cluster**		**BMI**	**BMI mini**	**Ageonset**	**Duration prog**.	**No.hospit**.	**EDQOL**				**EAT**			**Morgan and Russel**				
							**psy**	**Phy cog**	**end**	**Work sch**	**Dieting**	**Bulimia**	**Control**	**A**	**B**	**C**	**D**	**E**
α	m	14.7	13.8	17.4	3.6	1.3	1.5	1.7	3.4	2.7	21.6	8.1	10.1	1.0	2.5	5.9	7.1	7.2
*n* = 88 (61.5%)	SD	1.4	1.3	4.7	3.8	1.5	0.7	0.8	0.7	0.9	9.8	3.8	4.3	1.4	4.2	1.6	2.3	1.9
β	m	13.6	12.4	15.3	3.8	1.7	2.3	2.6	3.8	3.0	7.5	2.6	6.1	3.0	3.8	6.0	7.9	7.8
*n* = 31 (21.7%)	SD	1.0	1.2	3.6	3.5	1.7	0.9	0.7	0.3	0.9	6.4	2.7	3.7	2.0	5.0	1.8	2.0	2.4
**γ**	m	13.3	11.7	15.0	7.9	7.8	1.1	1.5	3.0	1.9	27.4	10.0	11.0	0.6	0.3	4.8	5.7	5.2
*n* = 24 (16.8%)	SD	1.2	0.9	3.9	5.7	7.9	0.5	0.7	1.2	0.9	8.1	3.5	4.3	1.3	1.6	1.7	2.2	2.1
δ	m	14.4	13.4	16.8	3.6	1.4	1.7	1.9	3.5	2.8	17.9	6.7	9.0	1.5	2.8	5.9	7.3	7.4
*n* = 119 (83.2%)	SD	1.4	1.4	4.7	3.7	1.6	0.9	0.9	0.7	0.9	10.9	4.3	4.5	1.8	4.4	1.6	2.3	2.0
Total	m	14.2	13.1	16.5	4.4	2.5	1.6	1.9	3.4	2.6	19.5	7.2	9.4	1.4	2.4	5.8	7.0	7.0
*n* = 143 (100%)	SD	1.4	1.5	4.5	4.4	4.3	0.8	0.9	0.8	0.9	11.1	4.3	4.5	1.8	4.2	1.7	2.3	2.2

*BMI, Body Mass Index; EAT, Eating Attitudes Test; EDQOL, Eating Disorder Quality of Life; psy, psychological; phy cog, physical/cognitive; end, financial; work sch, professional/academic; m, mean; SD, standard deviation; %, percentage*.

#### Classification in Three Clusters

##### Description of the α, β, and γ Clusters

The results are described in the Table 4. The γ cluster had the greatest severity profile of the three. It grouped patients that had average subscores on the GOAS supporting a more severe clinical expression of AN. As such, the eating dimension (A) was significantly affected; menstrual periods were the most altered (B); and the impact was most serious on the patient's mental state (C), psychosexual functioning (D), and socioeconomic status (E). The other assessment criteria also favored a more severe clinical expression of AN because this cluster was associated with the poorest nutritional state (BMI on admission and minimum BMI), the youngest age of AN onset, and the longest illness duration of the disorder, and the number of hospitalizations. The eating symptoms measured by the EAT scale were greater in the three subscales. The QOL was lower in all dimensions (EDQOL).

The α and β clusters had a more moderate severity profile than γ on all the variables. The α had variables that were mostly closer to β than γ with a slightly greater severity than β. However, α and β differed along two lines.

The α cluster showed a better nutritional status (higher BMI and minimum BMI) than the β and γ clusters. The age at onset was 2 years older on average; the illness duration and the number of hospitalizations were slightly lower. Conversely, the ED symptoms were very severe, close to those of the γ cluster (EAT score and GOAS subscore A).

The β cluster had a more severe nutritional impact (as measured by BMI and minimum BMI) than the α cluster. This can be explained by the fact that the α cluster assembled a significant majority of binge-purging type patients (AN-BP = 52, i.e., 59.1%), who normally have a better nutritional status and greater eating symptoms; conversely, the β cluster accounted for more restrictive type patients (AN-R = 23, i.e., 74.2%; χ^2^ = 10.2, *p* = 0.001). In addition, the β-cluster patients had a mean maximum BMI that was the lowest of the three groups (max BMI = 18.5 kg/m^2^) and those of the α cluster had the highest (max BMI = 20.9 kg/m^2^). In the β cluster, the illness duration and the number of hospitalizations were slightly higher. The age of onset was close to that of the γ cluster. Therefore, the severity seemed gradual: moderate for the α cluster (with the exception of the food symptoms) and β cluster (with the exception of the nutritional status), increased for all the criteria of the γ cluster. There was no significant difference in weight loss between the three clusters (maximum BMI over the lifetime minus admission BMI).

##### Comparison of Severity Profiles Based on the Existence of a Comorbidity

The prevalence of MDD and GAD comorbidities differed significantly according to the clusters. For these two disorders, the γ cluster was associated with more frequent comorbidities compared with the β cluster (75% MDD over the lifetime for patients of the γ cluster vs. 35.7% for the β cluster; OR = 5.4 [1.5–19.3]; 65% GAD over the lifetime for patients of the γ cluster vs. 17.9% for the β cluster). In addition, the prevalence of GAD was greater compared with the α cluster (α = 24.7%, OR = 5.7 [2.0–16.4]; β = 17.9% and γ = 65.0%, OR = 8.5 [2.3–32.4]). The prevalence distribution of OCD and social phobia was not significantly different between the clusters (Supplementary Table B).

#### Classification Into Two Clusters

##### Description of the δ and γ Clusters

When the sample was classified into two clusters, the α and β clusters were assembled to form the **δ** cluster. The **γ** cluster had more severe characteristics in all areas than the **δ** cluster (see Table 4).

##### Comparison of the Severity Profiles Based on the Existence of a Comorbidity

MDD and GAD in a lifetime were associated significantly more frequently with a profile of major severity (respectively, *p* = 0.03 and *p* < 0.001) (γ cluster). This same tendency was also noted for social phobia (*p* = 0.063) (Table 5).

**Table 5:** Comparison comorbidity frequencies based on 2 (δ and γ) clusters (Pearson's χ^2^ test).

	**Cluster**		**χ^**2**^**	***p***	***Post-Hoc p* < 0.05**
**Comorbidity**	**δ**	**γ**			
MDD	49 (48.5%)	15 (75%)	4.7	**0.030**	**δ < γ**
OCD	22 (21.8%)	7 (35.0%)	1.6	0.20	
GAD	23 (22.8%)	13 (65.0%)	14.2	**<0.001**	**δ < γ**
SP	27 (26.7%)	9 (45.0%)	5.5	0.06	

*MDD, Major depressive disorder; OCD, Obsessive-compulsive disorder; GAD, Generalized anxiety disorder; SP, Social Phobia. Bold values represents significant results*.

Comparison of severity profiles based on the chronology of onset of the comorbidities or their absence (Supplementary Table C).

The γ cluster had GAD associated with concomitant or later appearance relative to AN that was significantly different from the δ cluster.

## Discussion

The aim of this study was to investigate if the existence of an AD disorder comorbidity over a lifetime, especially when it started before AN, is associated with a more severe clinical expression of AN in the acute phase of the disorder.

Our main results confirm the first part of our initial hypothesis, namely, that patients with AN in the acute phase had greater overall severity (GOAS) when they experienced a comorbid disorder of MDD, GAD, or social phobia in their lifetime. When we tested the relationship between these three severity profiles and the comorbidities, we noticed that they differed for the prevalence of GAD and MDD. More specifically, the profile of maximum severity (γ) was associated with a significantly higher prevalence of MDD and GAD than one of the two profiles of lesser severity (β cluster). This later had the distinction of a more severe nutritional profile, with a major proportion of AN-R compared with the other (α). The solution in 2 clusters maintained this same difference (between γ and the δ cluster, which grouped together the two other profiles α and β).

On the other hand, the chronology of onset of the comorbidity or its absence was usually not associated with greater severity of AN regardless of the comorbid disorder. Most of the observed differences contrasted the absence of comorbidity against comorbidity before and/or after AN. The only differences observed were with MDD or GAD in which the age of onset of AN was later in the case of a comorbidity prior to AN compared with a later onset.

There is some evidence that emotional dysregulation, including anxiety and depression, are associated with poorer decision making in AN (32, 33). According to Bechara, decision making inherently relies on emotional processes that provide important implicit and explicit knowledge by which the individual is able to make fast and adaptive decisions (34). Van Elburg explains that “these emotional processes guide decision-making on several levels, including *via* bioregulatory processes, such as somatic marker signals, and occur both consciously and outside of awareness” (35). The literature highlights that an “affect-driven belief system profoundly influences the transformation of action into choices” and proposes that affect in particular plays a role in decision making that involves uncertainty, which is similar to the type of decision making mostly studied in AN (32, 35, 36). The severity of ED symptoms that we observed in conjunction with AD comorbidities could be explained by the link between anxiety and depression with poorer food decision making in AN.

### Severity and Presence of a Comorbidity

The population of our study had frequent comorbidities (over a lifetime and concomitant), which is in accordance with the data in the literature. In our sample, the prevalence of MDD over the entire life was 57% (37) and that of GAD was 32.2% (5). This is considerable for a sample with a mean age of 20 years as the mean age of onset of GAD in the general population is 25 years (38). The prevalence of OCD varies in the literature between 10 and 62% (5, 39, 40); our sample falls within this interval (27%). The literature found a prevalence equivalent to that in our sample for social phobia, 32.2% (5, 37).

In addition, the patients had major severity criteria in all aspects: overall condition (GOAS), eating symptoms (EAT), mean BMI, and a very altered QOL. This homogeneity is associated with the sample under consideration; it is a sample of hospitalized patients except that the admission criteria are generally based on a highly deteriorated clinical state (41).

Our univariate analyses showed that, when patients experienced a comorbidity of MDD, GAD, or social phobia in their lifetime, the clinical severity of AN, in terms of general clinical condition, eating symptoms, and QOL was significantly greater than in the absence of these comorbid disorders. As the prevalence of OCD was lowest in the sample, the sample sizes were limited, and our analyses lacked power for this disorder; this might explain why no significant difference was found on the GOAS for OCD although the results went in this direction. As cited above, we studied AN in general without differentiating the *restrictive* (AN-R) subgroups or those *with binge eating, vomiting, or use of purgatives* (AN-BP) although several studies associate a higher prevalence of OCD with the restrictive subtype (38–40).

With regard to the severity of the disorder in terms of continuum of care, comorbid GAD was associated with both an average illness duration that was two times longer than for patients without a disorder (2.9 vs. 6.2 years) and more frequent hospitalizations. The duration of progression in the group with GAD is considerable. The average illness duration in a clinical population is 4 years (10). These hospitalizations and the duration of disorders considerably affect the subject's life, contribute to their social isolation, and create a real vicious circle, which perpetuates the disorder and increases complications (42). GAD was also associated with a poorer nutritional state over the lifetime (minimum BMI), and a minimum BMI in the course of life that is very low (<13) is a factor of excess mortality (43). The BMI on admission did not vary significantly based on the existence of a comorbidity as certain authors have already noted (44, 45).

When the relationship between the severity profiles established from AN assessment components is studied, the cluster of extreme severity (γ) was associated with a prevalence of MDD that was 5.4 times higher in comparison with β, and seven and 8.5 times higher for GAD over a lifetime in comparison with the less severe α and β profiles, respectively. The distribution of frequencies of OCD and social phobia did not differ significantly among the clusters.

### Severity and Chronology of Onset of the Comorbidity

Our hypothesis, which was that AN would be more severe when the comorbid disorders occurred before rather than after or were non-existent, was only verified for MDD or GAD. In univariate analyses, the age of onset of AN was higher when MDD and GAD occurred before AN. The other severity indices did not differ based on the chronology or absence of a disorder. A later age of onset was described as a poor outcome factor in terms of mortality (4, 11, 46). When the ages of onset of MDD and GAD were studied, they were found to be significantly lower in patients whose disorder started before the AN (MDD-before = 13.8 years and GAD-before = 11.3 years) compared with those whose disorder started concomitantly or after the AN (MDD-after = 16.5 years and GAD-after = 16.3 years). The age of onset of disorders before the ED were very early compared with the typical age of onset of these disorders (around 25 years for both of them) (38); this is in accordance with the literature, which shows that anxiety disorders in childhood are known risk factors for the development of EDs (47).

### Limitations of the Study

One of the study limitations is the estimation of the onset and the illness duration (AN and comorbid disorders), which was undertaken retrospectively. The chronology of onset is calculated from these data and is, therefore, imprecise, especially because the diagnoses of proven comorbidity, remission, and recovery include a time dimension (for example, MDD is considered cured after a complete remission phase of 4–6 months).

We performed many statistical comparisons owing to the diversity of the chosen assessment parameters. AN is a disorder with a complex clinical expression; assessing its severity can be performed using certain methods. The DSM-5 proposes graduating the severity only according to the BMI for adults or the percentile of BMI for children and adolescents. Based on this criterion, our sample is in the extreme category because the mean BMI was below 15 (14.3; SD: 1.5). As remarked by Machado et al. (48), this single criterion is not a good reflection of the overall severity of AN because it does not take into account the pathophysiology of the specific expression of symptoms of the ED, its developmental profile or its impact on daily life. We, therefore, explored all these dimensions in this study. Despite the large number of statistical tests performed, however, we have not made any correction on the comparisons. As recommended by Bender and Lange (49), we did not suggest adjusting the alpha level (multiple comparisons) because this study was an exploratory step; its design was not intended to test causal hypotheses. Recruitment took place in second- or third-line centers after failure of management. This explains the long duration of development as well as the number of hospitalizations observed. Our population was, therefore, not comparable to all anorexic patients, which limits the generalization of our results to less severe patients. Despite this selection, however, we were able to demonstrate severity profiles in the subjects related to the comorbidity.

## Conclusion, Prospects for Research, and Clinical Practice

All of these factors support our understanding that greater severity of the disorder on admission is associated with an increased prevalence of MDD and GAD. This severity is a risk factor for chronicity, somatic, and psychiatric morbidity and mortality (4). Huas et al. (50) have already found some of our criteria of severity to be factors associated with excess mortality, including an older age, long progression of the disorder, and more severe eating symptoms. As a result, it is important to look for a history of MDD or GAD at the hospital admission in the most severe patients. Early treatment of these disorders during hospitalization, particularly with psychotropics, could perhaps facilitate an improvement in their prognosis (5, 51). This early treatment is currently not put into practice because the present guidelines (41) recommend refeeding before introducing treatments. Indeed, an aspect of AD symptoms relates to malnutrition and its treatment through refeeding (12). This phenomenon has led some authors to suggest that anxiety disorders during AN are only “artifacts” related to malnutrition. Furthermore, these recommendations are also based on the fact that antidepressant treatments have not been proven to be effective on AN or on the occurrence of depression (52). However, no therapeutic trial has yet evaluated the impact of early treatment and targeting MDD and GAD in cases of severe AN. This could be of interest to the extent that these comorbid psychiatric disorders are linked to clinical severity, and they impact the duration of hospitalization and probably the subsequent prognosis in terms of morbidity and mortality given the associated clinical severity (53).

## Data Availability Statement

The raw data supporting the conclusions of this article will be made available by the authors, without undue reservation.

## Ethics Statement

The studies involving human participants were reviewed and approved by Ile-de-France III Ethics Committee and the CNIL (Commission nationale de l'informatique et des libertés). Written informed consent to participate in this study was provided by the participants' legal guardian/next of kin. In accordance with the Helsinki declaration, written informed consent was obtained from each patient before inclusion, and from the parents of those who were under 18 years old. Both classes of patient (either adults or children and their parents) gave written consent.

## Consent For Publication

We confirm that the manuscript has been read and approved by all named authors and that there are no other persons who satisfied the criteria for authorship but are not listed. We further confirm that the order of authors listed in the manuscript has been approved by all of us. We confirm that we have given due consideration to the protection of intellectual property associated with this work and that there are no impediments to publication, including the timing of publication, with respect to intellectual property. In so doing we confirm that we have followed the regulations of our institutions concerning intellectual property.

## Author Contributions

AR and NG: design of the study. ER, NG, LM, SB, CH, FH, CL, and JD: revision of the manuscript. ER, AR, NG, LM, SB, CH, FH, and CL: article writing and revising. AR and NG: data analysis. All authors approved the submitted version.

## EVHAN Group

Nathalie Godart^1,2^, Sylvie Berthoz^1,2^, Christophe Lalanne^2^, Jeanne Duclos^1,2,3^, Lama Mattar^1,2^, Hélène Roux^1,2^, Marie Raphaële Thiébaud^1,2^, Sarah Vibert^1,2^, Tamara Hubert^2^, Annaig Courty^1,4^, Damien Ringuenet^4^, Jean-pierre Benoit^1,5^, Corinne Blanchet^1,5^, Marie Rose Moro^1,5^, Laura Bignami^6^, Clémentine Nordon^6^, Frédéric Rouillon^6,7^, Solange Cook^8^, Catherine Doyen^6,8^, Marie-Christine Mouren Siméoni^6^, Priscille Gerardin^9^, Sylvie Lebecq^9^, Marc-Antoine Podlipski^9^, Claire Gayet^9^, Malaika Lasfar^9^, Marc Delorme^10^, Xavier Pommereau^10^, Stéphanie Bioulac^10,11^, Manuel Bouvard^10,12^, Jennifer Carrere^10^, Karine Doncieux^13^, Sophie Faucher^13^, Catherine Fayollet^13^, Amélie Prexl^13^, Stéphane Billard^14,15^, François Lang^14,15^, Virginie Mourier-Soleillant^14^, Régine Greiner^14^, Aurélia Gay^14,15^, Guy Carrot^14,15^, Sylvain Lambert^16^, Morgane Rousselet^16,17^, Ludovic Placé^16,17^, Jean-luc Venisse^16,17^, Marie Bronnec^16,17^, Bruno Falissard^18^, Christophe Genolini^19^, Christine Hassler^18^, Jean-Marc Tréluyer^20^, Olivier Chacornac^20^, Maryline Delattre^20^, Nellie Moulopo^20^, Christelle Turuban^20^ and Christelle Auger^20^. Lead author of the EVHAN Group: Nathalie Godart (nathalie.godart@fsef.net)

^1^CESP, INSERM, University Paris-Sud, UVSQ, University Paris-Saclay, Paris, France

^2^Institut Mutualiste Montsouris, Paris, France

^3^University of Reims, EA 6291, Reims, France

^4^Hospital Paul Brousse, AP-HP, Villejuif, France

^5^Maison de Solenn, Hospital Cochin, AP-HP, Paris, France

^6^CMME, Saint Anne Hospital, Paris, France

^7^INSERM Center 894, Paris, France

^8^Hospital Robert Debré, AP-HP, Paris, France

^9^University Hospital of Rouen, France

^10^University Hospital of Bordeaux, Bordeaux, France

^11^USR CNRS 3413 SANPSY, Bordeaux, France

^12^University Victor Ségalen Bordeaux 2, Bordeaux, France

^13^Institut Marcel Rivière, La Verrière, Le Mesnil Saint-Denis, France

^14^University Hospital of Nord, Saint-Etienne, France

^15^University of Saint-Etienne, EA 4556 laboratory Epsylon, France

^16^University Hospital of Nantes, Nantes, France

^17^University of Nantes, EA 4275, France

^18^CESP, INSERM, University Paris-Sud, UVSQ, University Paris-Saclay, Villejuif, France

^19^UMR 1027, Toulouse, France

^20^URC-CIC Cochin Necker, AP-HP, Paris, France.

## Conflict of Interest

The authors declare that the research was conducted in the absence of any commercial or financial relationships that could be construed as a potential conflict of interest.
