# Erratum: Is the Severity of the Clinical Expression of Anorexia Nervosa Influenced by an Anxiety, Depressive, or Obsessive-Compulsive Comorbidity Over a Lifetime?

**DOI:** 10.3389/fpsyt.2021.781375

**Published:** 2021-10-18

**Authors:** Frontiers Production Office

**Affiliations:** Frontiers Media SA, Lausanne, Switzerland

**Keywords:** anorexia nervosa, anxiety, depression, nutritional status, body mass index

Due to a production error, there was an error in the order of the authors. The EVAN group should be listed as the 8th author.

The publisher also wishes to correct the affiliations for Nathalie Godart. The city for affiliation 7 was also corrected to Paris. The corrected author and affiliation lists appear below.

Elise Riquin^1,2,3†^, Agathe Raynal^4†^, Lama Mattar^5^, Christophe Lalanne^6^, France Hirot^7,8^, Caroline Huas^7,9^, Jeanne Duclos^10,11^, EVHAN group, Sylvie Berthoz^8,12‡^ and Nathalie Godart^7,8,9,13*‡^

^1^ Department of Child and Adolescent Psychiatry, Centre Hospitalier Universitaire d'Angers [Angers University Hospital], Angers, France

^2^ Laboratory of Psychology, LPPL EA4638, University of Angers, Angers, France

^3^ Adolescent and Young Adult University Hospital Department of the Health Foundation of Students of France, Centre Pierre Daguet, Sablé-sur-Sarthe, France

^4^ Department of Child and Adolescent Psychiatry, CH du Rouvray-CHU de Rouen, Rouen, France

^5^ Nutrition Division, Department of Natural Sciences, School of Arts and Sciences-Lebanese American University, Beirut, Lebanon

^6^ Université Paris Diderot [Paris Diderot University], Paris Sorbonne Cité, Paris, France

^7^ CESP, Univ. Paris-Sud, UVSQ, INSERM U 1178, Université Paris-Saclay [Paris-Saclay University], Paris, France

^8^ Department of Psychiatry for Adolescents and Young Adults, Institut Mutualiste Montsouris, Paris, France

^9^ Adolescent and Young Adult University Hospital Department of the Health Foundation of Students of France, Paris, France

^10^ Univ. Lille, CNRS, CHU Lille, UMR 9193—SCALab—Cognitive and Affective Sciences, Lille, France

^11^ Hôpital Saint Vincent de Paul, GHICL, Département de Psychiatrie, Paris, France

^12^ Univ. Bordeaux INCIA CNRS UMR 5287, Bordeaux, France

^13^ UFR des Sciences de la Santé Simone Veil [Simone Veil Health Science Training and Research Unit], Université de Versailles Saint-Quentin-en-Yvelines [Versailles Saint-Quentin-en-Yvelines University], Versailles, France

The publisher apologizes for this mistake. The original article has been updated.

