# Supplementary material for: Is the Severity of the Clinical Expression of Anorexia Nervosa Influenced by an Anxiety, Depressive, or Obsessive-Compulsive Comorbidity Over a Lifetime?
Source: Front Psychiatry. 2021 Jul 1;12:658416. doi: 10.3389/fpsyt.2021.658416 (PMC8280337; doi:10.3389/fpsyt.2021.658416)
Supplement: Supplementary file 1 [file Table_1.DOCX]

Supplementary material

Table A: Comparison of the means of AN clinical severity criteria based on the chronology of onset of the comorbid disorder or its absence (by ANOVA)

| Chronology of onset of the disorder relative to the start of AN | Absence of disorder (Abs) | Disorder before AN (Before) | Disorder concomitant to or after AN  (After) | *p* | *Post Hoc*  *p <0.05* |
| --- | --- | --- | --- | --- | --- |
|  | MR | | | |  |
| MDD | 5.3 | 4.4 | 4.4 | **<0.001** | Abs>Before; Abs>After |
| OCD | 4.8 | 5.1 | 4.3 | 0.16 |  |
| GAD | 5.0 | 4.3 | 4.5 | **0.03** | Abs>Before |
| Social phobia | 4.9 | 4.7 | 4.1 | 0.08 |  |
|  | BMI (kg/m2) | | | |  |
| MDD | 14.5 | 14.2 | 14.1 | 0.40 |  |
| OCD | 14.3 | 14.4 | 14.3 | 0.898 |  |
| GAD | 14.3 | 14.6 | 13.9 | 0.302 |  |
| Social phobia | 14.3 | 14.2 | 14.6 | 0.766 |  |
|  | BMI (kg/m2) | | | |  |
| MDD | 13.4 | 13.0 | 12.8 | 0.07 |  |
| OCD | 13.2 | 13.2 | 12.8 | 0.44 |  |
| GAD | 13.4 | 13.1 | 12.3 | **0.008** | Abs>After |
| Social phobia | 13.1 | 13.0 | 13.4 | 0.75 |  |
|  | Age of onset of AN | | | |  |
| MDD | 16.03 | 17.87 | 15.23 | **0.009** | **Before>After;** Before>Abs |
| OCD | 16.36 | 16.40 | 15.19 | 0.482 |  |
| GAD | 16.22 | 17.98 | 14.28 | **0.009** | **Before>After**; Abs>After |
| Social phobia | 16.43 | 15.97 | 15.37 | 0.603 |  |
|  | Duration of progression (years) | | | |  |
| MDD | 3.22 | 4.38 | 4.48 | 0.214 |  |
| OCD | 3.70 | 4.29 | 4.43 | 0.714 |  |
| GAD | 2.95 | 5.25 | 6.84 | **<0.001** | Before>Abs; After>Abs |
| Social phobia | 3.68 | 4.05 | 4.14 | 0.868 |  |
|  | Number of hospitalizations | | | |  |
| MDD | 1.85 | 3.95 | 2.71 | **0.042** | Before>Abs |
| OCD | 2.45 | 2.26 | 3.83 | 0.394 |  |
| GAD | 1.94 | 4.50 | 3.70 | **0.008** | Before>Abs; After>Abs |
| Social phobia | 2.49 | 3.10 | 2.40 | 0.762 |  |
|  | EAT | | | |  |
| MDD | 30.76 | 41.49 | 37.55 | **0.002** (26.7%) | Before>Abs; After>Abs |
| OCD | 32.88 | 42.00 | 42.41 | **0.006** | Before>Abs; After>Abs |
| GAD | 32.71 | 41.00 | 41.33 | **0.010** | Before>Abs; After>Abs |
| Social phobia | 31.60 | 42.85 | 46.38 | **<0.001** | Before>Abs; After>Abs |
|  | EDQOL | | | |  |
| MDD | 2.51 | 2.08 | 2.23 | **0.002** | Abs>Before; Abs>After |
| OCD | 2.37 | 2.14 | 2.07 | **0.050** | Abs>After |
| GAD | 2.39 | 2.14 | 2.16 | 0.084 |  |
| Social phobia | 2.42 | 1.98 | 2.15 | **0.001** | Abs>Before |

Legends: Before: comorbidity before AN onset, After: comorbidity after AN onset, and Abs: absence of comorbidity: MDD: Major depressive disorder; OCD: Obsessive-compulsive disorder; GAD: Generalized anxiety disorder; EAT: Eating Attitudes Test; EDQOL: Eating Disorder Quality of Life
