# Supplementary material for: Is the Severity of the Clinical Expression of Anorexia Nervosa Influenced by an Anxiety, Depressive, or Obsessive-Compulsive Comorbidity Over a Lifetime?
Source: Front Psychiatry. 2021 Jul 1;12:658416. doi: 10.3389/fpsyt.2021.658416 (PMC8280337; doi:10.3389/fpsyt.2021.658416)
Supplement: Supplementary file 2 [file Table_2.DOCX]

Table B: comparison of comorbidity frequencies based on clusters (Pearson’s Chi2 test)

|  | Cluster | | | χ2 | p | Post Hoc  p<0.05 |
| --- | --- | --- | --- | --- | --- | --- |
| comorbidity | **α** | **β** | **γ** |  |  |  |
| MDD | 39 (53.4%) | 10 (35.7%) | 15 (75%) | 7.3 | **0.02** | **β < γ** |
| OCD | 19 (26.0%) | 3 (10.7%) | 7 (35.0%) | 4.2 | 0.12 |  |
| GAD | 18 (24.7%) | 5 (17.9%) | 13 (65.0%) | 14.7 | **0.001** | **α < γ; β < γ** |
| SP | 23 (31.5%) | 4 (14.3%) | 9 (45.0%) | 5.5 | 0.06 |  |

Legends: MDD: Major depressive disorder; OCD: Obsessive-compulsive disorder; GAD: Generalized anxiety disorder; SP: Social phobia; EAT: Eating Attitudes Test; EDQOL: Eating Disorder Quality of Life
