# Supplementary material for: Is the Severity of the Clinical Expression of Anorexia Nervosa Influenced by an Anxiety, Depressive, or Obsessive-Compulsive Comorbidity Over a Lifetime?
Source: Front Psychiatry. 2021 Jul 1;12:658416. doi: 10.3389/fpsyt.2021.658416 (PMC8280337; doi:10.3389/fpsyt.2021.658416)
Supplement: Supplementary file 3 [file Table_3.DOCX]

Table C: Comparison of the chronology of onset of comorbidities or their absence based on the two clusters (Pearson’s Chi2 test)

| Chronology of onset of the  disorder relative to AN |  | Cluster | | χ2 | p | Post Hoc  p<0.05 |
| --- | --- | --- | --- | --- | --- | --- |
|  |  |  | |  |  |  |
|  |  |  | |  |  |  |
|  |  | δ | γ |  |  |  |
| GAD | concomitant or  after AN | 9 (9.1%) | 7 (36.8%)* | 15.18 | **0.001** | δ < γ |
|  | before AN | 12 (12.1%) | 5 (26.3%)* |  |  | δ < γ |
|  | Absence | 78 (78.8%) | 7 (36.8%) |  |  | δ > γ  Abs>After  Abs>Before |

* theoretical sample less than 5

Legends : AN : Anorexia Nervosa ; GAD : Generalized Anxiety Disorder
